# Analysis of Implicit Communication of Motorists and Cyclists in Intersection Using Video and Trajectory Data

**DOI:** 10.3389/fpsyg.2022.864488

**Published:** 2022-04-25

**Authors:** Meng Zhang, Mandy Dotzauer, Caroline Schießl

**Affiliations:** ^1^Institute of Transportation Systems, German Aerospace Center (DLR), Berlin, Germany; ^2^Institute of Transportation Systems, German Aerospace Center (DLR), Braunschweig, Germany

**Keywords:** vulnerable road users, implicit communication, intersection, right-turn, evaluation

## Abstract

The interaction of automated vehicles with vulnerable road users is one of the greatest challenges in the development of automated driving functions (ADF). In order to improve efficiency and ensure the safety of mixed traffic, ADF need to understand the intention of vulnerable road users, to adapt to their driving behavior, and to show its intention. However, this communication may occur in an implicit way, meaning they may communicate with vulnerable road users by using dynamic information, such as speed, distance, etc. Therefore, investigating patterns of implicit communication of human drivers with vulnerable road users is relevant for developing ADF. The aim of this study is to identify the patterns of implicit communication of human drivers with vulnerable road users. For this purpose, the interaction between right-turning motorists and crossing cyclists was investigated at a traffic light controlled urban intersection. In the scenario, motorists and cyclists had a green signal at the same time, but cyclist had right-of-way. Using the Application Platform for Intelligent Mobility (AIM) Research Intersection, trajectory and video data were recorded at an intersection in Braunschweig, Germany. Data had been recorded for 4 weeks. Based on the criticality metric post-encroachment time (PET) and quality of the recorded trajectory, 206 cases of interaction were selected for further analyses. According to the video annotation, when approaching the intersection, three common communication patterns were identified: (1) no yield, motorists, who should yield to cyclists, crossed the intersection first while forcing right-of-way; (2) active yield, motorists, who were in front of cyclists, gave the right-of-way; (3) passive yield, motorists, who were behind cyclists, had to give the right-of-way. The analysis of the trajectory data revealed different patterns of changes in time advantage in these three categories. Additionally, the communication patterns were evaluated with regard to frequency of occurrence, efficiency, and safety. The findings of this study may provide knowledge for the implementation of a communication strategy for ADF, contributing to traffic efficiency as well as ensuring safety in the interaction with vulnerable road users.

## Introduction

Communication between road users is an essential part of road traffic. In order to improve efficiency and ensure road traffic safety, road users need to understand the intention of other road users, to adapt to their driving behavior, and to show its intention. From the perspective of motorists, this communication can involve a series of explicit information, such as, facial expressions, gestures, eye contact, visual signs, or acoustic signals (Risser, 1985). However, recent studies revealed that pedestrians used vehicles’ movement (e.g., speed and acceleration) rather than explicit communication cues to decide whether it is safe to cross (Dey and Terken, 2017; Lee et al., 2020). Moreover, in the self-driving future, automated driving functions (ADF) may be required to communicate its intention by using dynamic information. The interpersonal communication may be eventually replaced by a human-machine interface (HMI) that may mainly depend on the implicit cues. Therefore, to match prior experiences and expectations of the passengers and the surrounding road users (Bengler et al., 2020), it is relevant to investigate the patterns of implicit communication of human road users, particularly in safety-critical situations.

Communicating the intentions with each other is an essential part of a smooth cooperation, even in heavily regulated traffic situations (e.g., controlled by traffic lights, at which the right-of-way between crossing and turning traffic road users is regulated). One of the most common safety-critical scenario in the right-hand traffic is when motorized road users turn right at an intersection, while cyclists approach from the right side of the motorists and cross (Richter and Sachs, 2017). In this particular situation, motorists need to give right-of-way to cyclists. Mostly, motorists focused on the road that they planned to merge into and failed to observe right-of-way or failed to detect the cyclists (Polders et al., 2015). On the other hand, injured cyclists stated that they expected that motorists would give right-of-way, as this corresponded to the regulation (Räsänen and Summala, 1998). The results from the investigations and the crash analyses show that traffic regulation alone does not prevent critical situations between motorists and cyclists. For example, studies showed that motorists do not always give right-of-way to crossing pedestrians and cyclists (referred to as vulnerable road users; VRU) when leaving the roundabout, although the right-of-way of VRU is regulated (Räsänen and Summala, 1998; Silvano et al., 2015). The interpretation is that, in addition to traffic safety, individual time efficiency and comfort are also relevant, suggesting road users compromise between following the rules and individual preferences (Nygårdhs et al., 2020). This may be a challenge for ADF. While it needs to understand the current circumstance, incl. traffic regulations, infrastructure, and surrounding road users, it also needs to consider personal preferences without sacrificing safety.

The kinematic information of other road users or the temporal and spatial relationships between road users are usually considered as implicit communication cues. Fuest et al. (2018) conducted a field study investigating implicit communication in a shared space and suggested that pedestrians decide on whether to cross the road by observing changes in vehicle speed. Usually, deceleration is understood as “give way” (Beggiato et al., 2017; Ackermann et al., 2018). On the other hand, a higher velocity of the vehicle is not perceived as giving right-of-way to other road users (Himanen and Kulmala, 1988; Šucha, 2014; Silvano et al., 2015). Furthermore, time-proximity indicators are also considered as implicit communication cues. For example, post-encroachment time (PET) is an observed time describing the time interval by which two road users missed each other. Time advantage (TAdv) is used to predict the time that two road users would miss each other, if they would continue with the same speed and trajectories. In previous studies, TAdv was applied for risk estimation (Saul et al., 2021) and also used to indicate which road user temporarily dominates: A road user with larger TAdv probably passes first (Laureshyn et al., 2010). Additionally, road users’ decision may also rely on the physical distance from the junction. Assuming that TAdv is one second, compared with the road users, who are 10 meters away from the junction, those who are 100 meters away, may have a better chance to adjust their speed and trajectories.

A number of traffic safety studies involved implicit communication between motorists and vulnerable road users in intersections. In previous studies, implicit communication was usually divided into two categories (motorists yielding and motorists not yielding) in the light of which road user crosses first (Sakshaug et al., 2010; De Ceunynck et al., 2013; Silvano et al., 2015). Várhelyi (1998) generalized three categories of vehicle’s braking behavior (no braking, provoked braking, and ideal interactions), when approaching a zebra crossing. Furthermore, focusing on the yielding behavior, van Haperen et al. (2018) defined four types of crossing behavior: taking, getting, forcing, and receiving describing the most common implicit communication patterns between motorists and cyclists in intersections. However, the consideration of implicit communication processes as well as the evaluation of implicit communication patterns were rare.

The aim of this study is to reveal the implicit communication patterns by analyzing one of the most common safety-critical situations, right-turning motorist and crossing cyclist. The implicit communication patterns were described by analyzing road users’ behavior, particularly, from the perspective of motorists. The following research questions will be answered: What categories of implicit communication can be identified? What is the frequency of the categories of implicit communication? How does implicit communication effect efficiency and safety?

## Methods

### Infrastructure

**FIGURE 1:**
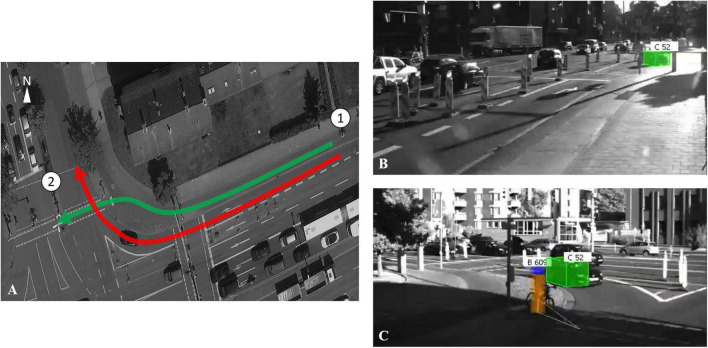
**(A)** Path of right-turning motorists (red) and crossing cyclists (green) as well as the positions of two poles. **(B)** The view of camera one. **(C)** The view of camera two.

As part of the Intelligent Mobility Application Platform (AIM), an infrastructural detection system was implemented at the intersection of Hagenring/Rebenring in Braunschweig (Knake-Langhorst et al., 2016). Two poles equipped with stereo cameras and infrared lighting were installed at the Western and Southern ford of the intersection enabling the detection of crossing cyclists and right-turning motorists in the Eastern arm when approaching the intersection (approx. 35–40 m) and the point of conflict at the intersection (see Figure 1). The output of the system is trajectory data with corresponding videos. The data from the two sensor systems were merged and processed in real time with a sampling rate of 25 Hz. The position of the road user in the Universal Transverse Mercator (UTM) system and the size and category of the road user were detected in the video. The corresponding trajectory data was derived from the video data. In addition, speed, acceleration, and heading of the road user were also derived by using the position of the road user. Road users were assigned to the following categories: cars, trucks, vans, cyclists, pedestrians. The video material of road users and events was anonymized in real time and saved with a low resolution so that neither license plates nor faces were recognized or tracked.

### Material

**FIGURE 2:**
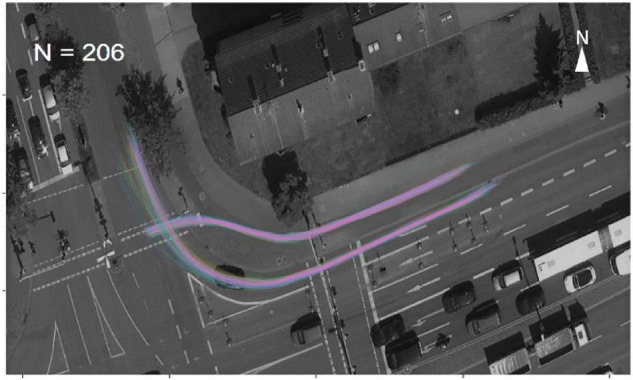
Trajectories of 206 selected cases.

The data had been recorded for 4 weeks: From August 22nd to September, 18th 2016. In order to find the valid interactions of right-turning motorists and crossing cyclists, the PET was used. According to previous studies (Svensson, 1998; Zangenehpour et al., 2016; Johnsson, 2020), interactions with a PET value of less than 1.5 s were considered as dangerous or very dangerous interaction. We chose a higher threshold (i.e., PET < 2.5 s) as we aimed at identifying a wide range of interaction behavior. Altogether, 1,201 interactions of turning motorists and crossing cyclists were initially selected as candidates. Additionally, in order to ensure the quality of the required data for subsequent analysis, we used a package of the density-based spatial clustering of applications with noise (DBSCAN, Hahsler et al., 2019) in the R programming language to cluster the valid paths and exclude cases that contained a high proportion of data outside the valid path. Poor detection may cause road users to appear outside the valid path. Particularly, road users may indeed appear outside the valid path, for instance, when cyclists travel on the sidewalk instead of the bicycle lane. Those cases were also excluded, since they were considered as not representative of normal cycling behavior. Thus, 206 cases of interaction were selected for further analyses (see Figure 2).

### Scenario

We focused on the scenario, in which motorists turned right from Hans-Sommer-Strasse into Brucknerstrasse, while cyclists crossed the intersection of Hans-Sommer-Strasse (see Figure 1A). In the scenario, motorists were on the right turn lane and cyclists on the protected bicycle lane. They had a green signal at the same time, but according to the local traffic regulations, cyclist had right-of-way. The data in the area from when both of the road users were detectable [about 35–40 meters (m) before the intersection] to when one of the road users exceeded the junction of two paths was considered as the valid data, in which the subsequent annotation and analysis were performed.

In the video annotation, changes in relative position of the right-turning motorist and the crossing cyclist was coded. Therewith, several common interaction patterns between motorists and cyclists were identified when approaching the intersection: For example, motorists were in front of cyclists at all times; motorists were in front, then abreast, and, in the end, behind cyclists; motorists stayed behind cyclists; motorists were behind, then abreast, and, again, behind cyclist.

In order to classify the annotated interaction patterns and give them a semantic meaning, we revised and used the classification of the yielding behavior proposed by van Haperen et al. (2018). According to the previously annotated changes in relative position, the following three categories were defined from the perspective of motorists:

• No yield: The motorist, who should yield to the cyclist, crosses the intersection first while forcing right-of-way (incl. the cases, when motorists were in front of cyclists in the end).
• Active yield: The motorist, who is in front of the cyclist, gives the right-of-way (incl. the cases, motorists were in front of cyclists at first and in the end behind cyclists).
• Passive yield: The motorist, who is behind the cyclist, gives the right-of-way (incl. the cases, motorists were abreast or behind cyclists at first and in the end behind cyclists).

In the original version of yielding behavior classification, a fourth category is proposed describing the situation in which the cyclist gives right-of-way through explicit communication cues (e.g., waving to a driver). Due to the low resolution, we could not identify the waving movement. Thus, receiving was not considered in the following analysis.

### Analysis

We investigated the communication categories, no yield, active yield, and passive yield, through the indicators of frequency of occurrence, efficiency, and safety dimension. We used relative frequency to indicate the frequency of occurrence of a communication category. Journey time and standard deviation (SD) of speed was used for efficiency of communication categories. Given that the detection range is consistent and the driving/riding range is limited to the road segment, the journey time is associated with the velocity. With regard to the safety analysis, PET and T2 was used. Additionally, the perspectives of different road users were also considered in the analysis (see details in Table 1).

**TABLE 1:** Description of Indicators, which were used in analysis.

Dimension	Indicator	Perspective	Description
Frequency	proportion (%)	Both	The proportion of categories in the defined scenario
Efficiency	Journey time [s]	Both	The time interval from when the one of the road users appears to when both leave the intersection
		Vehicle	The time interval from when the vehicle appears to when it leaves the intersection
		Bicycle	The time interval from when the bicycle appears to when it leaves the intersection
	Standard deviation (SD) of speed (m/s)	Vehicle	Standard deviation of vehicle speed
		Bicycle	Standard deviation of bicycle speed
Safety	PET (s)	Both	Post-encroachment time of the interaction between two road users.
	T2 (s)	Both	The arriving time of the second (later) road user, at the moment when the first road user arrives at the crossing point.

According to the results of Shapiro–Wilk normality tests, journey time, SD of vehicle speed, PET, and T2 were not normally distributed [journey time of both: the statistic of Shapiro–Wilk tests (*W*) = 0.94, *p*-value (*p*) < 0.001; journey time of vehicle: *W* = 0.96, *p* < 0.001; journey time of bicycle: *W* = 0.96, *p* < 0.001; SD of vehicle speed: *W* = 0.98, *p* < 0.05; PET: *W* = 0.88, *p* < 0.001; T2: *W* = 0.90, *p* < 0.01]. Therefore, we used Kruskal–Wallis tests as well as pairwise Wilcoxon-Tests (with Holm method for adjusting *p*-values) to analyze the effect of communication categories on journey time, PET, and T2, respectively. The results were converted into *Z*-score. To determine the effect size of Kruskal–Wallis tests, the parameter η*^2^* recommended by Tomczak and Tomczak (2014) was used. Hereby, the effect size is low when η*^2^* less than 0.06, medium when η*^2^* less is than 0.14 and large when η*^2^* is greater than 0.14. One-way ANOVA was applied to exam the effect of communication categories on SD of bicycle speed. Additionally, we used pairwise *t*-tests (with Holm method for adjusting *p*-values) to compare between categories. For significant effects, the Cohen’s *f* was provided, where 0.1, 0.25, and 0.4 represent low, medium, and large effect size, respectively. The significance level of α = 0.05 was used for the overall test.

## Results

### Description of Implicit Communication Patterns

**FIGURE 3:**
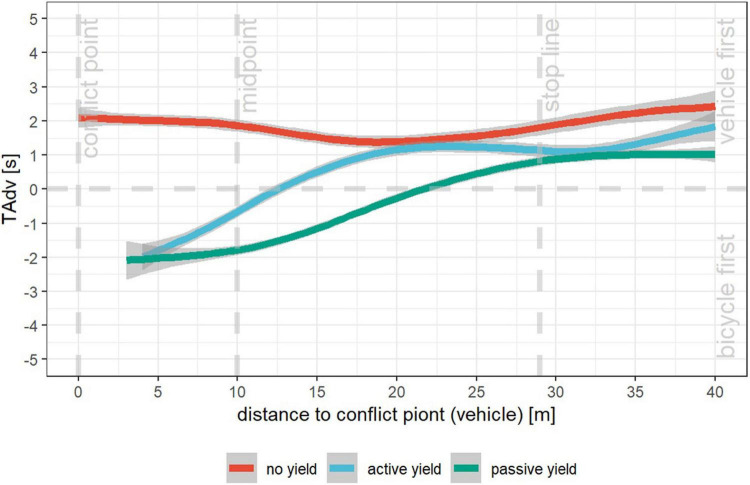
Averaged TAdv in no yield (red), active yield (blue), and passive yield (green) on vehicle’s distance to conflict point.

In summary, 206 interactions between right-turning motorists and crossing cyclists were annotated. According to the changes in relative position, they were classified into three categories: no yield, active yield, and passive yield. Figure 3 shows the different implicit communication processes of these three categories by using averaged TAdv on vehicle’s distance to conflict point, meaning that the predicted time, that two road users would miss each other, was averaged within the category at each point. In Figure 3, before the stop line, results of TAdv indicate that independent of the corresponding category, vehicles are generally in front of bicycles and are supposed to cross first, if both of the road users maintain their speed and trajectories. At the stop line and at the 20 m before conflict point, motorists in passive yield and in active yield start to lose their advantage. On the contrary, motorists in no yield lead the way and the TAdv is almost always above 1 s. According to the changes in TAdv, the three communication categories present completely different patterns. However, they have one thing in common: the second road user (cyclist in no yield, motorist in active yield and passive yield) always crossed with a time gap of approx. 2 s.

### Frequency of Occurrence

In 177 (86%) cases, cyclists crossed the intersection before motorists, while only 29 (14%) motorists crossed the intersection first. Additionally, according to their relative position, the cases were classified into three categories: no yield (29, 14%), active yield (103, 50%), and passive yield (74, 36%).

### Efficiency

**FIGURE 4:**
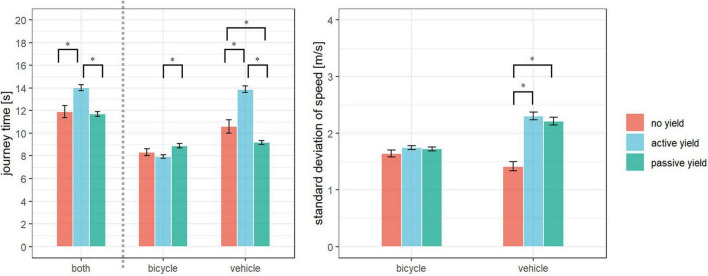
Mean and standard error of journey time from the perspective of both road users, bicycle and vehicle (left) as well as standard deviation of speed of bicycle and vehicle (right) in no yield (red), active yield (blue), and passive yield (green) (*p < 0.05).

From the perspective of both road users, the Kruskal–Wallis tests indicated significant differences in journey time across the communication categories no yield, active yield, and passive yield (*Z* = 6.25, *p* < 0.001, η*^2^* = 0.2). According to the pairwise comparisons using Wilcoxon tests (α = 0.05), the journey time of both road users in active yield [median (Mdn) = 13.56 s, interquartile range (IQR) = 2.82 s] was significantly greater than in no yield (Mdn = 11.4 s, IQR = 2.92 s, *Z* = 3.51, *p* < 0.001) and in passive yield (Mdn = 11.24 s, IQR = 2.12 s, *Z* = 5.97, *p* < 0.001). There was no difference between the journey time of both road users in no yield and passive yield (*Z* = 1.71, *p* = 0.96). From the perspective of the motorists, a significant difference was observed between the categories no yield, active yield, and passive yield (*Z* = 10.11, *p* < 0.001, η*^2^* = 0.52). According to the results of the Wilcoxon tests (α = 0.05), the journey time of vehicle in active yield (Mdn = 13.48 s, IQR = 3.06 s) was significantly greater than in no yield (Mdn = 10.56 s, IQR = 4.24 s, *Z* = 4.61, *p* < 0.001) and in passive yield (Mdn = 9.16 s, IQR = 1.93 s, *Z* = 10.01, *p* < 0.001). The journey time of vehicle in no yield was greater than in passive yield (*Z* = 2.04, *p* < 0.05). From the perspective of bicycle, a significant difference was observed between the categories no yield, active yield, and passive yield according to the Kruskal–Wallis tests (*Z* = 2.91, *p* < 0.001, η*^2^* = 0.05). The journey time of bicycle was greater in passive yield (Mdn = 8.58 s, IQR = 2.26 s) than in active yield (Mdn = 7.84 s, IQR = 1.54 s, *Z* = 2.78, *p* < 0.001). There was no difference between passive yield and no yield (Mdn = 7.96 s, IQR = 1.56 s, *Z* = 0.91, *p* = 0.18) and between active yield and no yield (*Z* = 0.02, *p* = 0.51) (see Figure 4).

The Kruskal–Wallis tests indicated a significant difference in SD of vehicle speed between the communication categories (*Z* = 5.78, *p* < 0.001, η*^2^* = 0.18). According to the pairwise comparisons using Wilcoxon tests (α = 0.05), the SD of vehicle speed in no yield (Mdn = 1.44 m/s, IQR = 0.4 m/s) was significantly less than in active yield (Mdn = 2.21 m/s, IQR = 1.17 m/s, *Z* = 5.43, *p* < 0.001) and in passive yield (Mdn = 2.23 m/s, IQR = 0.8 m/s, *Z* = 5.38, *p* < 0.001). There was no difference between the SD of vehicle speed in active yield and passive yield (*Z* = 0.06, *p* = 0.48) (see Figure 4).

The one-way ANOVA indicated no significant difference in SD of bicycle speed between the communication categories [*F*(2,203) = 1.29, *p* = 0.28, *f* = 0.11]. The mean SD of bicycle speed in no yield, active yield, and passive yield were 1.64 m/s (SD = 0.33 m/s), 1.74 m/s (SD = 0.32 m/s), and 1.72 m/s (SD = 0.29 m/s), respectively (see Figure 4).

### Safety

**FIGURE 5:**
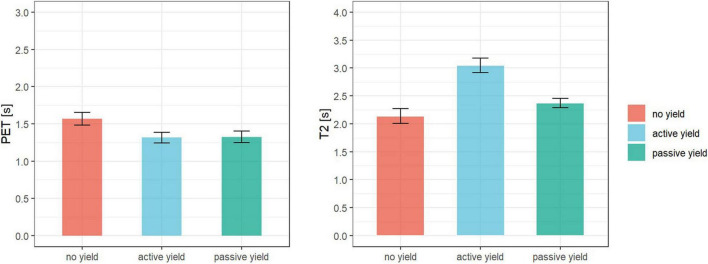
Mean and standard error of PET (left) T2 (right) in no yield (red), active yield (blue), and passive yield (green).

The median PET of no yield, active yield, and passive yield were 1.48 s (IQR = 0.64 s), 1.2 s (IQR = 0.8 s), and 1.26 s (IQR = 0.55 s), respectively (see Figure 5). The Kruskal–Wallis test indicated a significant difference in PET between the communication categories (*Z* = 2.35, *p* < 0.01, η*^2^* = 0.03). According to the pairwise comparisons using Wilcoxon tests (α = 0.05), the PET in no yield was significantly greater than in active yield (*Z* = 1.98, *p* < 0.05) and in passive yield (*Z* = 1.96, *p* < 0.05). There was no difference between the PET of active yield and passive yield (*Z* = 0.16, *p* = 0.56).

The Kruskal–Wallis test indicated a significant difference in T2 between the communication categories (*Z* = 4.14, *p* < 0.001, η*^2^* = 0.09). According to the pairwise comparisons using Wilcoxon tests (α = 0.05), the T2 in active yield (Mdn = 2.77 s, IQR = 1.52 s) was significantly greater than in no yield (Mdn = 1.97 s, IQR = 0.91 s, *Z* = 3.35, *p* < 0.001) and in passive yield (Mdn = 2.25 s, IQR = 0.91 s, *Z* = 3.07, *p* < 0.001). There was no difference between the T2 of no yield and passive yield (*Z* = 1.42, *p* = 0.08) (see Figure 5).

## Discussion

The aim of this study was to reveal implicit communication patterns between human drivers and VRU. Three implicit communication patterns from the perspective of motorists, no yield, active yield, and passive yield, were identified by analyzing the interaction between right-turning motorists and crossing cyclists. Additionally, frequency of occurrence, efficiency, and safety were analyzed in order to gain knowledge about the performance of the implicit communication patterns. The no yield communication pattern has the lowest probability of occurrence, while active yield occurred more often than passive yield and no yield. Active yield with a higher journey time may suggest more time-consuming interactions. Lower SD of vehicle speed in no yield may be interpreted as less variant and more stable travel through the intersection, which could be considered as more efficient from the perspective of motorists. With regard to the safety analysis, we used PET and T2 to reveal the (prospective) time that two road users missed each other. For both indicators, a lower value may suggest a more critical encounter (Svensson, 1998). Higher PET values in no yield implies a safer interaction, while active yield appears to be safer than passive yield and no yield, because the second road user provided a larger time distance (T2).

According to our analysis, these three implicit communication patterns (i.e., no yield, active yield, and passive yield) represent an interaction strategy when right turning motorists and crossing cyclists approach an intersection. Modeling common interpersonal interactions may help ADF to have a proper interpretation of each other’s behaviors (Ezzati Amini et al., 2019). One of the most important aspects is to understand that decisions on driving maneuvers are affected by temporal and spatial characteristics. As mentioned previously, road users who are 10 m away and 100 m away may have different alternatives when facing an encounter with TAdv of one second. In our cases, the directionless changes of TAdv, between 30 and 40 m (in passive yield) 20 and 30 m (in no yield and active yield) away from conflict point, show the hesitation of road users implying the underlying negotiation. On the other hand, the monotone increase in TAdv between 0 and 20 m (in no yield), the monotone decrease in TAdv between 0 and 20 m (in active yield), the and monotone decrease in TAdv between 0 and 30 m (in passive yield) may indicate that road users negotiate in the correspondent section (see Figure 3). The results may suggest that the section between 20 and 30 m ahead of the crossing point is relevant for the road users for communication and decision process.

The evaluation of human road users’ implicit communication may improve the humanization of ADF. The 86% yield rate provides *a priori* probability for autonomous driving functions when turning right at an intersection. Furthermore, the passive to active yield rate of approx. 7:10 suggests a frequency of common yielding behavior of human driver. This may help ADF to minimize the impact on common interpersonal interactions. According to the German traffic law, motorists need to yield to cyclists when turning right in an intersection. Thus, ADF is supposed to brake on its own initiative in order to yield to cyclists actively. Furthermore, the active yield is considered as the safer interaction, particularly from the perspective of cyclists, which was also proven in previous study (Várhelyi, 1998). But if motorists are well ahead of cyclists and already in the process of turning, they may probably cross before the cyclist. The 29 no yield cases proved the existence of this situation. No yield situations may be interpreted as a trade-off of motorists’ individual efficiency and safety, since both, the journey time of motorist (which oppositely indicates efficiency) and the T2 (which indicates safety) in no yield, are lower than the other two patterns. However, the severity of the injuries in a potential collision should not be neglected. The lower journey time of the vehicles resulted in higher speed leading to an increased severity as well as a higher risk. Thus, further research needs to take into account indicators of severity (e.g., Delta-V; Laureshyn et al., 2017) to improve the definition of margins of safety.

The evaluation of human road users’ implicit communication may help ADF to understand the intention of VRUs. Compared with passive yield, active yield with a lower cyclists’ journey time may suggest less time-consuming interactions. In the result-oriented interpretation, it could be treated as a cooperative behavior, namely, motorists sacrificed their own efficiency to improve the efficiency of cyclists or cyclists sped up to reduce waiting times for motorists. However, it was noted that the difference in efficiency exists only between the cyclists’ journey time of active yield and passive yield. The cyclists’ journey time and SD of cyclist speed did not appear to be impacted by implicit communication patterns. On the one hand, most cyclists in these cases may not change their crossing behavior, since they may intend to take the regulated right-of-way. On the other hand, the implicit communication patterns (no yield, active yield and passive yield) were classified from the perspective of motorists neglecting the scenarios, where cyclists obviously reacted to motorists. Therefore, fine classification of implicit communication patterns from the both perspective of road users is needed in the further research.

The major limitation of this research is that the influencing factors, such as, traffic flow and the number of conflicts were not considered. According to a recent study (Wu and Xu, 2017), high traffic flow may lead to a sharper deceleration when approaching the intersection. Furthermore, it was inferred that drivers are more likely to yield, when more than two pedestrians are crossing the intersection. The categorization of implicit communication would be more robust if the influencing factors such as traffic flow and the number of crossing VRUs could be controlled. Correspondingly, it also means that a larger sample is required.

A simulator study may be considered alternatively since it provides a controllable experimental environment compared with the naturalistic driving setting. Additionally, it may provide the opportunity to optimize the classification of implicit communication patterns using subjective reports of communication strategies. In our next steps, we will build up the identical setting of the intersection in the virtual environment and ask participants to drive or ride in the connected simulators in order to verify the categorization of implicit communication.

## Conclusion

This research reveals patterns of implicit communication of motorists with cyclists using video and trajectory data. Furthermore, the communication patterns were evaluated with regard to frequency of occurrence, efficiency, and safety. The results of this research may improve the humanization of ADF.

## Data Availability Statement

The raw data supporting the conclusions of this article will be made available by the authors, without undue reservation.

## Author Contributions

MZ, MD, and CS: conceptualization, methodology, and writing – review and editing. MZ: data curation, visualization, and writing – original draft. MZ and MD: investigation. CS: project administration. CS and MD: supervision. All authors contributed to the article and approved the submitted version.

## Conflict of Interest

The authors declare that the research was conducted in the absence of any commercial or financial relationships that could be construed as a potential conflict of interest.

## Publisher’s Note

All claims expressed in this article are solely those of the authors and do not necessarily represent those of their affiliated organizations, or those of the publisher, the editors and the reviewers. Any product that may be evaluated in this article, or claim that may be made by its manufacturer, is not guaranteed or endorsed by the publisher.

## References

[B1] Ackermann C. Beggiato M. Bluhm F. Krems J. (2018). “Vehicle Movement and its Potential as Implicit Communication Signal for Pedestrians and Automated Vehicles,” in *Proceedings of the 6th HUMANIST Conference*, eds Van Nes N. Voegelé C. (Lyon: Humanist Publications), 86–92. 10.3389/frobt.2022.818019

[B2] Beggiato M. Witzlack C. Krems J. F. (2017). “Gap Acceptance and Time-To-Arrival Estimates as Basis for Informal Communication between Pedestrians and Vehicles,” in *Proceedings of the 9th International Conference on Automotive User Interfaces and Interactive Vehicular Applications* AutomotiveUI ’17, (Oldenburg: Association for Computing Machinery), 50–57. 10.1145/3122986.3122995

[B3] Bengler K. Rettenmaier M. Fritz N. Feierle A. (2020). From HMI to HMIs: towards an HMI Framework for Automated Driving. *Information* 11:61. 10.3390/info11020061

[B4] De Ceunynck T. Polders E. Daniels S. Hermans E. Brijs T. Wets G. (2013). Road Safety Differences between Priority-Controlled Intersections and Right-Hand Priority Intersections: behavioral Analysis of Vehicle–Vehicle Interactions. *Transport. Res. Rec.* 2365 39–48. 10.3141/2365-06 12716185

[B5] Dey D. Terken J. (2017). “Pedestrian Interaction with Vehicles: Roles of Explicit and Implicit Communication,” in *Proceedings of the 9th International Conference on Automotive User Interfaces and Interactive Vehicular Applications*, (Oldenburg: ACM), 109–113. 10.1145/3122986.3123009

[B6] Ezzati Amini R. Katrakazas C. Antoniou C. (2019). Negotiation and Decision-Making for a Pedestrian Roadway Crossing: A Literature Review. *Sustainability* 11:6713. 10.3390/su11236713

[B7] Fuest T. Michalowski L. Träris L. Bellem H. Bengler K. (2018). “Using the Driving Behavior of an Automated Vehicle to Communicate Intentions - A Wizard of Oz Study,” in *2018 21st International Conference on Intelligent Transportation Systems (ITSC)*, (Maui: IEEE). 3596–3601. 10.1109/ITSC.2018.8569486

[B8] Hahsler M. Piekenbrock M. Doran D. (2019). dbscan: fast Density-Based Clustering with R. *J. Stat. Softw.* 91 1–30. 10.18637/jss.v091.i01

[B9] Himanen V. Kulmala R. (1988). An application of logit models in analysing the behaviour of pedestrians and car drivers on pedestrian crossings. *Accident Analys. Prevent.* 20 187–197. 10.1016/0001-4575(88)90003-6 3382496

[B10] Johnsson C. (2020). *Surrogate Measures of Safety with a Focus on Vulnerable Road Users: An Exploration of Theory, Practice, Exposure, and Validity.* [PhD thesis]. Lund: Lund University.

[B11] Knake-Langhorst S. Gimm K. Frankiewicz T. Köster F. (2016). Test Site AIM – Toolbox and Enabler for Applied Research and Development in Traffic and Mobility. *Transport. Res. Proc.* 14 2197–2206. 10.1016/j.trpro.2016.05.235

[B12] Laureshyn A. De Ceunynck T. Karlsson C. Svensson Å Daniels S. (2017). In search of the severity dimension of traffic events: extended Delta-V as a traffic conflict indicator. *Accident Analys. Prevent.* 98 46–56. 10.1016/j.aap.2016.09.026 27690148

[B13] Laureshyn A. Svensson Å Hydén C. (2010). Evaluation of traffic safety, based on micro-level behavioural data: theoretical framework and first implementation. *Accident Analys. Prevent.* 42 1637–1646. 10.1016/j.aap.2010.03.021 20728612

[B14] Lee Y. M. Madigan R. Giles O. Garach-Morcillo L. Markkula G. Fox C. (2020). Road users rarely use explicit communication when interacting in today’s traffic: implications for automated vehicles. *Cogn. Tech. Work* 23 367–380. 10.1007/s10111-020-00635-y

[B15] Nygårdhs S. Kircher K. Johansson B. J. E. (2020). Trade-offs in traffic: does being mainly a car driver or a cyclist affect adaptive behaviour while driving and cycling? *Eur. Transp. Res. Rev.* 12:12. 10.1186/s12544-020-0396-y

[B16] Polders E. Daniels S. Hermans E. Brijs T. Wets G. (2015). Crash Patterns at Signalized Intersections. *Transport. Res. Rec.* 2514 105–116. 10.3141/2514-12 12716185

[B17] Räsänen M. Summala H. (1998). Attention and expectation problems in bicycle–car collisions: an in-depth study. *Accident Analys. Prevent.* 30 657–666. 10.1016/S0001-4575(98)00007-4 9678219

[B18] Richter T. Sachs J. (2017). Turning accidents between cars and trucks and cyclists driving straight ahead. *Transport. Res. Proc.* 25 1946–1954. 10.1016/j.trpro.2017.05.219

[B19] Risser R. (1985). Behavior in traffic conflict situations. *Accident Analys. Prevent.* 17 179–197. 10.1016/0001-4575(85)90020-X 4096785

[B20] Sakshaug L. Laureshyn A. Svensson Å Hydén C. (2010). Cyclists in roundabouts—Different design solutions. *Accident Analys. Prevent.* 42 1338–1351. 10.1016/j.aap.2010.02.015 20441851

[B21] Saul H. Junghans M. Dotzauer M. Gimm K. (2021). Online risk estimation of critical and non-critical interactions between right-turning motorists and crossing cyclists by a decision tree. *Accident Analys. Prevent.* 163:106449. 10.1016/j.aap.2021.106449 34749268

[B22] Silvano A. P. Ma X. Koutsopoulos H. N. (2015). When Do Drivers Yield to Cyclists at Unsignalized Roundabouts?: empirical Evidence and Behavioral Analysis. *Transport. Res. Rec.* 2520 25–31. 10.3141/2520-04 12716185

[B23] Šucha M. (2014). *Road Users’ Strategies and Communication: Driver-Pedestrian Interaction.* Available online at: https://trid.trb.org/view/1327765 (Accessed on Jan 16, 2022).

[B24] Svensson Å (1998). *A Method for Analysing the Traffic Process in a Safety Perspective.* [PhD thesis]. Lund: Lund University.

[B25] Tomczak M. Tomczak E. (2014). The need to report effect size estimates revisited. An overview of some recommended measures of effect size. *Trends Sport Sci.* 21 19–25. 10.1186/s13054-016-1208-6 27885969 PMC5493079

[B26] van Haperen W. Daniels S. De Ceunynck T. Saunier N. Brijs T. Wets G. (2018). Yielding behavior and traffic conflicts at cyclist crossing facilities on channelized right-turn lanes. *Transport. Res. F Traffic Psychol. Behav.* 55 272–281. 10.1016/j.trf.2018.03.012

[B27] Várhelyi A. (1998). Drivers’ speed behaviour at a zebra crossing: a case study. *Accident Analys. Prevent.* 30 731–743. 10.1016/S0001-4575(98)00026-8 9805516

[B28] Wu J. Xu H. (2017). Driver behavior analysis for right-turn drivers at signalized intersections using SHRP 2 naturalistic driving study data. *J. Saf. Res.* 63 177–185. 10.1016/j.jsr.2017.10.010 29203017

[B29] Zangenehpour S. Strauss J. Miranda-Moreno L. F. Saunier N. (2016). Are signalized intersections with cycle tracks safer? A case–control study based on automated surrogate safety analysis using video data. *Accident Analys. Prevent.* 86 161–172. 10.1016/j.aap.2015.10.025 26562673

